# Variant Creutzfeldt–Jakob disease surveillance in Spain, 1993–2021

**DOI:** 10.3389/fpubh.2026.1780793

**Published:** 2026-03-03

**Authors:** Jesús De Pedro-Cuesta, Fernando J. García López, Miguel Calero, María Ruiz-Tovar, Javier Almazán-Isla

**Affiliations:** 1 Department of Neurodegeneration, Mental Health and Ageing, National Epidemiology Centre, Carlos III Health Institute (ISCIII), Madrid, Spain; 2 Centro de Investigación Biomédica en Red sobre Enfermedades Neurodegenerativas, Madrid, Spain

**Keywords:** bovine spongiform encephalopathy (BSE), Creutzfeldt–Jakob disease, epidemiology, Spain, surveillance, transmissible spongiform encephalopathies (TSE), variant Creutzfeldt–Jakob disease (vCJD)

## Abstract

**Background:**

Variant Creutzfeldt–Jakob disease (vCJD) is a fatal transmissible prion disorder attributed primarily to the ingestion of meat from cattle infected with bovine spongiform encephalopathy (BSE), and it can also be transmitted through blood transfusions and occupational exposure in laboratories. As of July 2024, 233 deaths had been recorded in 12 countries, mostly in the United Kingdom (UK) and France.

**Objective:**

This study aimed to describe the results of vCJD surveillance in Spain from 1993 to 2021 and the responses to those results.

**Methods:**

Surveillance spanned the following three periods: 1993–2000, when neurologists and public health professionals created a network for reporting potential cases; 2001–2010, when diagnostic criteria were improved, and management guidelines were published; and 2011–2021, when reporting and post-mortem analyses decreased.

**Results:**

Five deaths due to vCJD were identified through post-mortem between 2005 and 2008 (ages ranging from 25 to 64 years). In two cases involving a mother and her son, their prion strain was similar to the strain found in UK humanized mouse models, suggesting a link to BSE. Three cases had recently undergone invasive dental procedures, one had undergone a fibroscopy, and another had donated blood several times. The majority of prevention measures were adopted with a delay.

**Conclusion:**

Spain is the third most affected country by vCJD. Interruption of beef imports from the UK and control of the local BSE epizootic may have helped control vCJD in Spain since 2009. The increase in vCJD deaths through occupational exposures in laboratory work in other countries since 2016 should act as a warning signal for the surveillance of all forms of human transmissible spongiform encephalopathies.

## Introduction

Variant Creutzfeldt–Jakob disease (vCJD) is a disorder caused by the aggregation and deposition of a pathologic isoform of the cellular prion protein (PrP^C^), denoted as PrP^Sc^. vCJD emerged clinically in the United Kingdom (UK) in 1994 and was first reported in 1996 (1). This disorder was not fully unexpected, as an *ad-hoc* surveillance system was set up in the UK in the early 1990s and later on in a few other EU member countries as a research activity to unveil potential consequences of dietary exposure to bovine spongiform encephalopathy (BSE) (2). vCJD garnered considerable attention and concern in Europe due to fears that the presence of the first patients could signal the beginning of an outbreak of unpredictable magnitude secondary to local BSE exposure after an incubation time of approximately 10 years. Twenty-five years later, as a result of surveillance and research regarding human transmissible spongiform encephalopathy (HTSE), the natural history of sporadic, genetic, and acquired prion disorders has been thoroughly reported, which provided a unique context for vCJD public health work in the 21st century (3).

vCJD, now a rare disorder due to significant public health interventions, has been extensively studied (4, 5). Dating back to 2 May 2022, 233 vCJD deaths had been recorded in 12 countries. Of these, the United Kingdom and France stand out, with 178 and 29 cases, respectively (6). In the UK, two persons with the M129V codon structure died with vCJD, one clinical case and a subclinical case in a person with hemophilia. The results of three national surveys of abnormal prion prevalence in archived appendix specimens showed that potential vCJD carriers may still exist (7). The etiological underlying factors appeared to be dietary exposure to BSE products by residence or travel to the UK, import of cattle raised in the UK or fed with meat and bone meal imported from the UK, as well as transmission by blood and blood products in a few cases (5). Recently, cases have been reported in laboratory workers, one in Italy, who died in 2016 (8), and two in France (9), who died in 2019 and 2021. In Spain, five cases have been reported during the 1993–2018 period, with three of them clustered by time and place of residence, and two of them were first-degree relatives with shared dietary habits (3, 10).

The purpose of this study was to describe three separate aspects of vCJD surveillance in Spain for the period 1993–2021: (1) surveillance procedures particularly relevant for vCJD, (2) surveillance outcomes for vCJD, and (3) response after surveillance, i.e., the preventive measures determined by the potential transmissibility of each vCJD case.

## Methods

As follows, we report on the public health structures and surveillance work relevant to vCJD in Spain.

Spain's population, 46 million in 2008 (the midpoint of the studied period), is served by a health care system that has an important public component, including pediatric and neurological diagnosis and care, as well as public health services progressively decentralized to 17 regions since 2001. However, it was an international network of researchers, rather than public health institutions, that took up the study of CJD in the early 1990s. Country-based CJD surveillance started in a few European Union (EU) member countries, formally as a Concerted EU Action coordinated from centers in Edinburgh, Scotland, and Rotterdam, The Netherlands, in the early 1990s, and crystalized as an extended EU public health network supported by the European Centre for Disease Prevention and Control as of 2007 (11). Spain joined the network in 1995. The basic features and outcomes of the Spanish HTSE surveillance system for the period 1993–2018 have recently been reported (3). Variation in the effectiveness of vCJD surveillance over the period probably existed, i.e., in part due to alarms in 1996 and 2001 secondary to the first vCJD cases in the UK and the BSE cases reported in Spain, respectively, as well as important technical advances in HTSE diagnosis introduced at specific time points and countries (3). An overall, structured view of surveillance methods during the 1993–2021 period is given in reference 3. The following is a summary of the characteristics mentioned in two publications from 2000 to 2010 covering the first two-thirds of the period (12, 13), and scientific reports on diagnostics (12).

### Period 1993–2000

**Figure 1:**
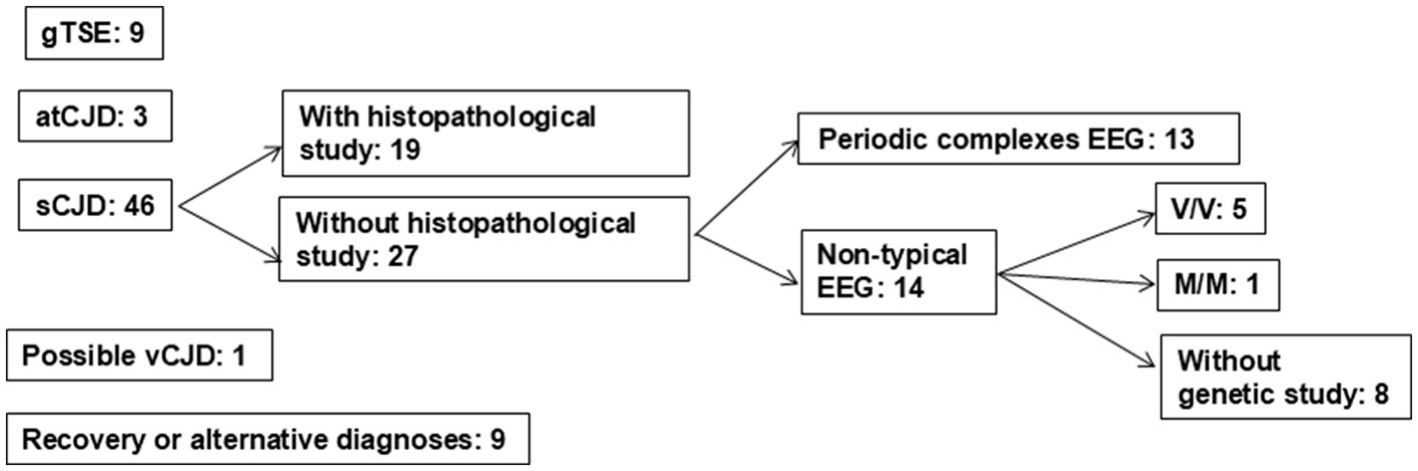
Suspected human transmissible spongiform encephalopathy in 70 patients under 56 years of age at onset in Spain from 1993 to 2003. Established final diagnoses and number. gTSE, genetic transmissible spongiform encephalopathy; atCJD, accidentally transmitted Creutzfeldt–Jakob disease; sCJD, sporadic Creutzfeldt–Jakob disease; vCJD, variant Creutzfeldt–Jakob disease; EEG, electroencephalogram; V/V, valine/valine; M/M, methionine/methionine. Modified from Grupo español de estudio y vigilancia de encefalopatías espongiformes (16).

The objectives of surveillance in Spain did not vary throughout this period. The aspects considered to be most relevant for vCJD surveillance in the first period were, first, the creation of a network of clinical neurologists integrated by a decentralized health services system with regional registers that retrospectively collected CJD diagnoses for 1993–1994. Subsequently, neurologists throughout the country prospectively identified and clinically evaluated suspected HTSE cases, with the assistance of a designated clinical coordinator knowledgeable about vCJD in each region; they reported suspected cases to regional public health officials, who in turn reported them to the National Epidemiology Centre, which reviewed and verified them centrally. The final classification was based on combined clinical, epidemiological, laboratory, and, when available, post-mortem neuropathological findings and was reviewed by the central coordination team. Missing or inconsistent information led to follow-up with regional coordinators; cases that remained insufficiently documented after repeated attempts were considered lost to follow-up according to predefined criteria. Throughout this period, attention was focused almost exclusively on epidemiological research, with limited attention paid to the control measures subsequently adopted. The increasing proportion of notifications with 14-3-3 brain protein in the CSF study, and MRI studies was evident (12). A few performed tonsil biopsies on immunohistochemically negative suspects. Four patients fulfilling the 2000 reported criteria (14, 15) with possible vCJD were notified. Two of them with clinical onset in 2000 or later had either an alternative diagnosis or a negative tonsil biopsy. Figure 1 shows the diagnoses and number of cases with suspected HTSE at age < 56 years (16). One case was diagnosed with possible vCJD and, among patients with non-typical electroencephalogram, eight did not undergo genetic study, and another without mutations was MM homozygous at the 129 codon. Notifications were made rather late, i.e., at advanced stages of the clinical course in 10%. Considerable improvements in HTSE ascertainment occurred during the period. A young Spanish national resident in the UK was diagnosed with vCJD in 1995 in Andalusia and reported to the UK.

### Period 2001–2010

The sensitivity of the system for the diagnosis of vCJD during this period improved, due in part to increased awareness among professionals and the media due to the impact of the five reported vCJD cases and the spread of the BSE epidemic. The quality of vCJD diagnoses improved with MRI, a powerful tool to reveal posterior thalamic lesions (17). Similarly, unlike the preceding 1993–2000 period, surveillance involved the systematic follow-up of HTSE suspects with a history of either temporary or permanent residence in a high BSE incidence area, the province of León, where three cases occurred. The 2010 annual report already mentioned a decrease in notifications.

Taking into account the growing concern by physicians and dentists about the safety of reusing medical instruments previously applied to patients with CJD (especially since 2006, following the first confirmed case of vCJD), the Ministry of Health convened a group of experts to agree on guidelines for diagnosis, management, evaluation, and prevention. Its objectives were to detect events identified by a routinely used protocol in vCJD suspects after analysis. In our study, in addition to describing vCJD cases, we provide reported incidents and the results of risk assessments of probable or definite vCJD cases and public health measures linked to specific vCJD cases. Most of them correspond to this central period.

### Period 2011–2021

The most remarkable characteristic in this period was the decrease in notifications during the last 6 years, 2016–2021, as well as a fall in the proportion of post-mortem examinations (222/529, 42%, in the period 2014–2018 vs. 28/180, 18%, in 2019-2021). This was mainly due to the loss of a national reference laboratory responsible for extracting brains from underserved regions, coupled with the disruption caused by the COVID-19 epidemic. An evaluation of the notifications made in 2012 (3) revealed problems in their follow-up, with losses of 12 of them. Criteria to declare the end of follow-up, i.e., definitely lost cases or case suspects, were established as late as 2017.

## Results

**Figure 2:**
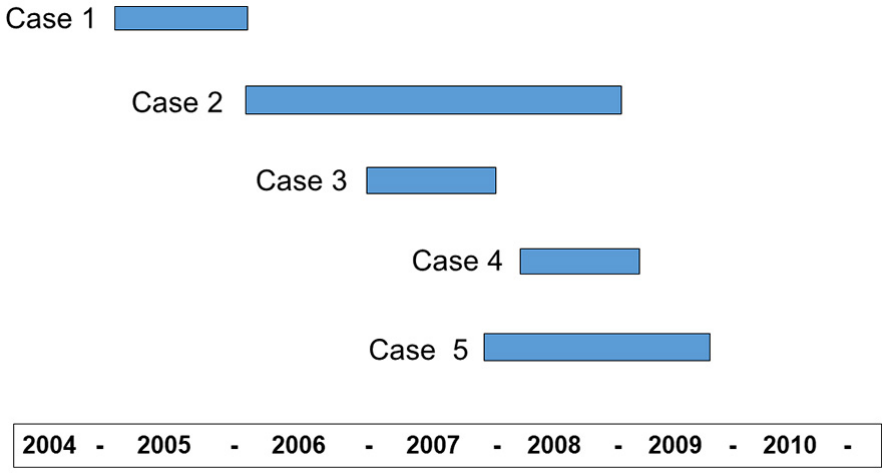
Clinical course during the period 2004–2010 of five patients with confirmed vCJD post-mortem.

As shown in Table 1, of 2,257 suspected cases reported during the period 1995–2021, five were diagnosed as post-mortem neuropathologically confirmed vCJD, while none of the others were ultimately classified as probable vCJD (Figure 2) (13). Table 1 summarizes the rather heterogeneous most relevant clinical and laboratory findings, including prion protein isoforms (18). There were three cases in the province of León, two of them mother and son with almost simultaneous clinical onset, and a third case, which had met the criteria of probable sCJD with triphasic EEG complexes, was finally diagnosed as vCJD after a detailed re-evaluation of her post-mortem examination. The age at clinical onset of confirmed vCJD cases ranged from 25 to 64 years, with a mean of 45.60 and a standard deviation (SD) of 13.95 years. Transmission to humanized mice with tissue from the two family-related cases showed, despite differences in patient age at clinical onset and disease manifestations, the same properties of the strain in these patients as in the UK vCJD cases (19).

**Table 1:** Demographic, clinical, and ancillary examination data for the five vCJD cases.

**Case no**	**1**	**2**	**3**	**4**	**5**
Sex	Female	Female	Male	Female	Female
Occupation	Laboratory technician	Administrative	Data technician	Housewife	Jewelery technician
Province	Madrid	León	León	León	Cantabria
Age at onset (years)	25	48	40	64	47
Month of clinical onset	November 2004	February 2006	May 2007	February 2008	August 2007
Death date	July 2005	December 2007	February 2008	August 2008	January 2009
Duration to death (months)	8	23	9	6	18
Symptoms at onset	Asthenia, painful dysesthesia in legs, dementia (memory loss, apathy, depression)	Personality & behavioral changes, apathy, depression, loss of olfactory and taste functions	Episodic diplopia, personality & behavioral changes with depression	Leg and muscle pain, anxiety, depression	Psychiatric symptoms, sleep and gait disorder, myoclonus
Additional symptoms profile	Difficulties with concentration and writing, cognitive deterioration with severe language disturbance Unsteadiness Increased startle response, Multifocal myoclonus	After several months: mild progressive, cognitive impairment with reading difficulties, self-care limitations, dysphagia requiring gastrostomy, myoclonus, gait disorder, akinetic mutism	After several months, disoriented in place & time, amnestic, language problems and rapid cognitive decline. After 6 months gait disturbance. Food inhalation	Dementia, parkinsonism, myoclonus	Dementia, myoclonus, akinetic mutism
Neurological status summary	At the long course (11 month): Spontaneously open eyes Inattentive Inconsistent responses to simple verbal commands Decreased verbal output Enhanced palmomental reflex Brisk myotatic reflexes Bilateral Babinski sign Multifocal myoclonus Unsteady stance and gait	At medium course (10 months): MMSE score 33/35 Decreased reasoning Phonetic paraphasia Bilaterally brisk reflexes Bilateral grasping Plantar extensor response Bilateral Hoffman's reflex Mild dysarthria Distal myoclonus	At the late course (9 months): Disoriented in time, place, and person, bradyphrenia, low verbal fluency, short-term amnesia, cognitive impairment with poor executive functions Unstable gait, ataxia Tremor and poor coordination of the upper extremities, dysarthria Bilateral pyramidal signs, myoclonus Akinetic mutism	At the late course (5 month): Unstable gait Cognitive impairment Insomnia Fluid dysphagia Altered postural reflexes Dysarthria Loss weight Hand trembling Bilateral pyramidal signs Myoclonus	At the late course (12 months): Cognitive impairment Apathy Confusion Disorientation Unstable gait Agnosia Myoclonus Akinetic mutism
14-3-3 protein in cerebrospinal fluid	Positive	Negative	Negative	Negative	Negative
*PRPN* gene	No mutation	No mutation	No mutation	No mutation	No mutation
Codon 129	Methionine/methionine	Methionine/methionine	Methionine/methionine	Methionine/methionine	Methionine/methionine
Electroencephalogram	Slow background rhythm	Periodic triphasic waves	Slow background rhythm	Slow background rhythm	Slow background rhythm
Magnetic resonance imaging	Bilateral pulvinar sign	Bilateral pulvinar sign	Bilateral pulvinar sign	Bilateral pulvinar sign	Bilateral pulvinar sign
Post-mortem study	Neuropathology, immunohistochemistry	Neuropathology, immunohistochemistry	Neuropathology, immunohistochemistry	Neuropathology, immunohistochemistry	Neuropathology, immunohistochemistry
Prion protein isoform (16)	Type 2B	Not tested	Type 2B	Type 2B	Type 2B

Numbers 3 and 4 were related, son and mother.

**Figure 3:**
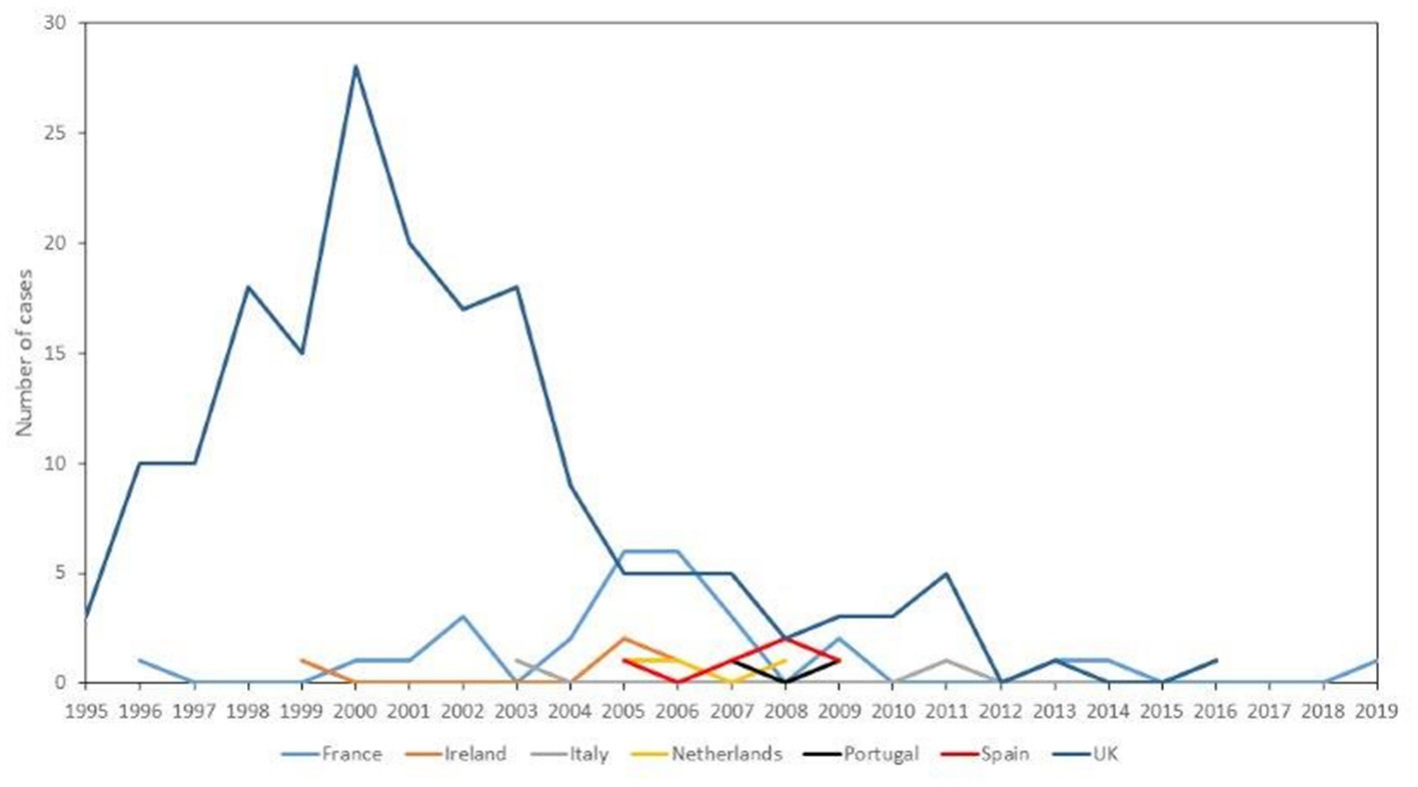
Number of vCJD cases in European countries between 1995 and 2019. vCJD, variant Creutzfeldt–Jakob disease.

Figure 3 shows the annual number of vCJD deaths in Spain together with the vCJD outbreaks in European countries during the period 1995–2019. vCJD in Spain appeared late after the UK and France epidemics, and the vCJD peak in France fits the first vCJD death in Spain. We lack information to interpret the temporal patterns of other countries.

Since 2009, no new vCJD has been identified in Spain for 15 years.

### vCJD risk factors recorded

The history of risk factors collected through a structured interview following the British model (20) was significant in a high proportion of cases, as shown in Table 2.

**Table 2:** History of potential risk factors and transmission risks.

**Case no**	**1**	**2**	**3**	**4**	**5**
Blood reception history	Negative	Negative	Negative	Negative	Negative
Dietary history	Systematic dietary exposure at workplace^*^	Not relevant	Brain or entrails (>1 per month)	Brain or entrails (>1 per month)	Not relevant
Occupational history	Fast food restaurant^*^, 1997–1999 Technician in a clinical analysis laboratory, 2000–2002 Technician in an animal health surveillance laboratory, 2000–2003	Not relevant	Not relevant	Not relevant	Not relevant
Surgical history Prior to clinical onset	None	Dental implants, 2002 Surgery for myopia correction, 2001 Left carpal nodule, 1987	Tonsillectomy, 1976	Sciatic nerve decompression, 2001 Varicose veins, 2004 Laryngoscopy, 2007	Endodontics, 2004
Blood donation	Yes^**^	No	No	No	No

^*^Restaurant associated with beef imports from the European Union.

^**^Blood or platelet transfusion for five people and industrial processing for coagulation factors.

Three of the five patients with vCJD had a history of etiologically relevant exposures. The first patient worked for 5–8 years prior to death in a restaurant linked to a company that occasionally imported meat from the European Union, and several people witnessed her frequent consumption of restaurant meals, including processed meat. For almost 4 years, until 2 years before the onset of her illness, she worked as a technician in an animal health surveillance laboratory focusing on fish. Regarding cases 3 and 4, according to relatives, the mother and her affected son used to eat beef brains and offal prepared by the mother at least once a month for a long time, except for a temporary interruption after 1995, when the news of the vCJD epidemic in the UK appeared.

Since the diagnosis of sporadic CJD in a neuropathologist in 2009, considerable attention has been paid to occupational exposures by healthcare workers in Spain. Although there is no clear evidence of occupational transmission, an increased occupational risk of CJD cannot be ruled out (21). A young patient with a long history of occupational exposure in HTSE research laboratories in Spain and two other EU member countries was reported in 2019, fulfilling criteria for probable sporadic CJD when he died in November 2022, although no post-mortem study was performed.

### Response to surveillance

We can define the response as the implementation of preventive actions aimed at limiting the spread of the disease after the identification of cases. Surveillance and response must be linked in an integrated system (22). In Spain, the regional health authorities are responsible for implementing response actions when vCJD cases are identified. In general, the central hub drafted a report or proposed coordinated action when incidents occurred. According to the guidelines established by the National Hemotherapy Commission regarding HTSE (23), blood products potentially contaminated with vCJD patients should have been withdrawn in Spain from 2001 onwards.

Table 2 shows some potential transmission risks reported in the vCJD cases. The most frequent incident was a visit to a dental clinic for treatment, which occurred in cases 2 and 5 a few years before the clinical onset of the disease. Regional health services or local hospitals designed preventive interventions in dental clinics where vCJD patients had been treated. These interventions were based on the guidelines for dentistry published by the central hub in 2006 (24).

As the first case donated blood annually between 1996 and 1999 inclusive, we reported the potential risk of transmission to the Ministry of Health. The regional blood transfusion center identified five recipients of blood or platelet concentrates, four of whom were deceased in August 2005 due to unrelated pathologies. The company that processed her blood specimens, both cells and plasma proteins and clotting factors, identified the contaminated materials and informed the Ministry of Health. It turned out that part of the patient's blood was included in batches used for the treatment of several hundred Spanish patients diagnosed with hemophilia, and another part of the blood derivative was sent to a Latin American country. Consequently, the Spanish Federation of Hemophilia Patients informed approximately 300 patients residing in the region of Madrid about the risk of transmission with the help of the Spanish Association of Hematology and Hemotherapy. The health authorities of the government of Madrid identified and monitored them. As of June 2024, no case from this group has been reported to the HTSE registry. At all times, the National Commission of Hemotherapy and the TSE Registry coordinated to monitor recipients and donors of blood components and derivatives.

Case 4 underwent a bronchoscopy without biopsy. Subsequently, up to 35 patients underwent fibroscopy with the same equipment. The central hub issued guidelines to the hospital's preventive service department with the procedures for washing and decontamination of the device, which was quarantined and then reassigned to non-clinical duties.

At the same time, several British citizens living in Spain were at risk of contracting vCJD due to blood transfusions from affected donors, generally during surgery procedures carried out in the UK. They were monitored for potential incidents and were reported to the British authorities.

To the best of our knowledge, none of the cases or their relatives received any compensation from the Spanish government or any other institution.

## Discussion

Surveillance of HTSE in Spain for more than 25 years shows that the five vCJD cases had particular characteristics. Compared to other countries, particularly the UK and France (1), Spain's peak calendar period, age at onset, and death, spatiotemporal clustering, family aggregation, and dietary habits showed some significant differences, with all the reservations stemming from the few affected cases. While the vCJD cases reported in the UK and France hardly differed in age and survival time, and the two cases in northern Portugal had a lower age of onset (25), the Spanish vCJD cases had a higher age at onset/death and a shorter survival time. On the other hand, some clinical parameters, such as the triphasic complex at EEG and a rapid course, mimicked sCJD characteristics.

**Figure 4:**
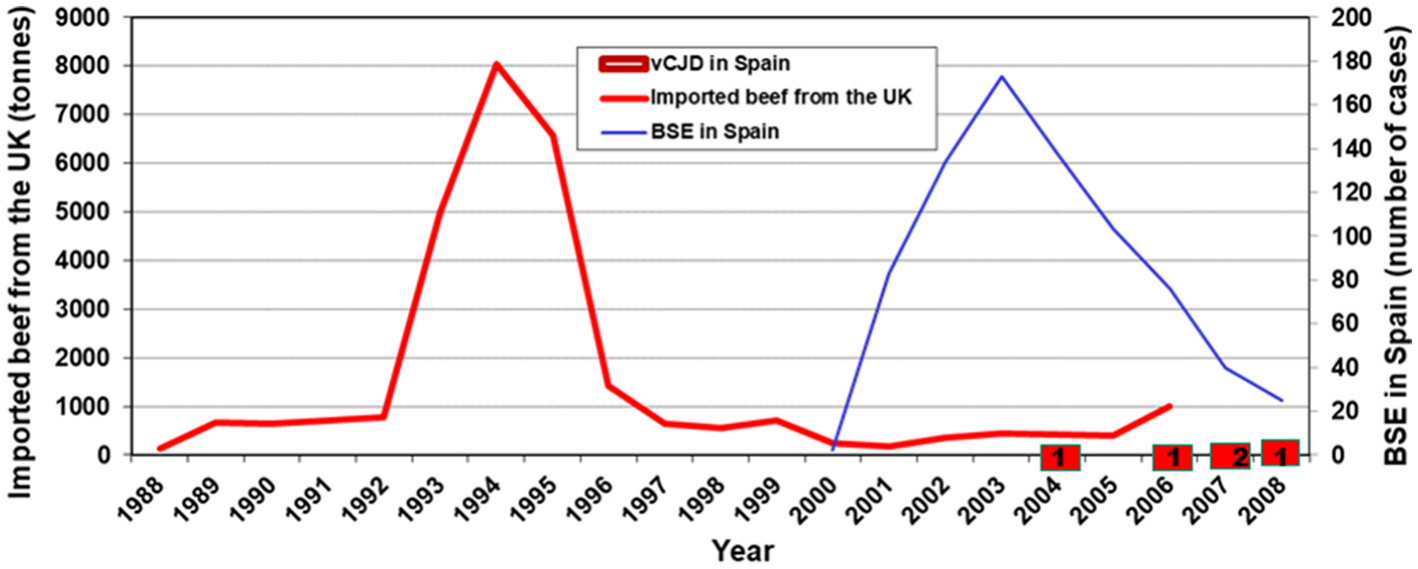
Imports of beef from the UK in tons between 1988 and 2008, annual number of BSE cases diagnosed in Spain during the same period and years of clinical onset of confirmed vCJD cases. UK, United Kingdom; vCJD, variant Creutzfeldt–Jakob disease.

To better understand the outbreak of vCJD cases in Spain, we must first consider the potential dietary exposures to BSE in Spain. Figure 4 shows the BSE exposure of the Spanish population, expressed by imports of beef from the UK, and the number of BSE cases diagnosed annually throughout the country and recorded up to 2010. Imports of beef from the UK into Spain started in 1993, peaked in 1995, and fell sharply to very low values in 1996, when vCJD was reported in the UK. The beef carcasses imported from the UK contained spinal cord. During the whole period, national annual beef production did not change over time (an annual average of 530 000 tons between 1989 and 1998) and was several hundred times higher than beef imports from the UK at any time. By contrast, the local BSE epidemic in Spain could have started in 2000, reached its peak in 2003, and almost disappeared in 2006. While beef consumption in Spain was almost constant, BSE dietary exposure appeared to have been limited and bimodal, first imported and then related to domestic BSE. The distribution of lesions found in the BSE cases in Spain suggested that the phenotype of the BSE strain found in the Spanish cases was the same as that reported in the British and Portuguese epidemics (26).

Based on ecological data and case reports from international surveillance, we could hypothesize etiological clues in a few cases. Approximately 10 years before the onset of case 1 (roughly the estimated incubation period for vCJD in the United Kingdom and France), imports of UK cattle carcasses containing spinal cord reached their peak in Spain, coinciding with the patient's occupational and dietary exposure. Her clinical profile, low age at onset, and particularly her dietary history might fit better with a causal exposure to imported BSE from the UK, a hypothesis for vCJD suggested in local BSE-free populations (27). The first vCJD case in the Netherlands, a 26-year-old woman at the time of her death with an occupational history of several years working and eating in a processed meat restaurant (28), resembles the first Spanish case, and both would fit the hypothesis of BSE meat imported from the UK.

For cases 3 and 4, the PrP strain present in the mother and son matched that seen in the UK humanized mouse models (19), suggesting a link between both cases and BSE arising in the UK. The clustering of cases 2, 3, and 4 in the province of León suggests that they shared a common etiology. The mother and son had a history of dietary exposure to beef brain and offal, potentially related to BSE, as it coincided with the onset of the BSE epizootic in Spain. The ultimate origin of BSE in León, where it was prevalent, could have been the meat and bone meal imported from Portugal, where UK exports were important. The province of León is close to northern Portugal, the region where two cases of vCJD were detected. Furthermore, in the province of León, domestic slaughtering of cattle and other animals existed at the turn of the century (Badiola J pers. comm.) and could have implicated cattle with BSE before BSE surveillance. Although the transmission of BSE from sheep in central Spain has been proposed (29), we believe that the León cluster is related to BSE. Unfortunately, we do not have any etiological clues regarding case 5 who is from Cantabria, a neighboring region to León. In summary, we suggest two etiological sources: one from beef with BSE imported from the UK (case 1) and one from local beef with BSE (cases 2, 3, and 4).

Prevention efforts derived from vCJD surveillance is a public health action distinct from food and feed control, and these efforts have been reflected in policies to increase safety in blood donation and the pharmaceutical industry (30), as well as in preventive measures implemented in hospitals. In response to surveillance results, we took measures to prevent the spread of the disease in three of the five cases (3). In order to ensure preventive action, this response requires an early, sustained, and strong commitment from national and regional institutions. However, we barely succeeded in this endeavor. The fact that at the time of diagnosis, patients may have transmitted the disease to others through their blood, fluids, or medical devices, due to the long incubation period before clinical onset, hinders prevention. Once a diagnosis of vCJD has been made, retrospective intervention is necessary to prevent further harm and to monitor all patients already exposed.

Our study has limitations. The surveillance system may have had low sensitivity to detect cases. Case 4 was initially misdiagnosed as probable sCJD. The post-mortem findings precluded this bias, but there were a few cases, especially in recent years, where post-mortem examinations were not performed, such as in the patient diagnosed with probable sCJD with a history of occupational laboratory exposure to prion disorders. Consequently, our surveillance might have underestimated the true number of cases. The clinical differences in the Spanish cases compared to cases from Portugal, France, and the UK, where young patients predominated, could have led to misdiagnosing other cases as sCJD. Another limitation has been the delay in reporting cases to the central hub, sometimes taking months, especially since 2020 with the COVID-19 pandemic, which could have hampered proper preventive and public health decision-making.

To recapitulate, we found five cases of vCJD in Spain during a short period of time, from 2005 to 2008. The absence of new vCJD cases since 2010 may primarily be due to the cessation of beef imports from the UK and effective control of the local BSE epidemic. We cannot rule out that response measures to surveillance might have helped to control vCJD. Due to the considerable plasticity of vCJD, the potential shift to an upward trend in the annual number of vCJD referrals determined by occupational exposures from laboratory research since 2016 (31) should act as a warning signal for surveillance of all forms of HTSE and encourage strategic causal research taking into account a broad view of rapidly progressing conformational proteinopathies.

## Data Availability

Access to data is restricted to protect personal information. However, confidential data will be available upon request to the corresponding author.
